# Comprehensive analysis of lncRNA-mRNAs co-expression network identifies potential lncRNA biomarkers in cutaneous squamous cell carcinoma

**DOI:** 10.1186/s12864-022-08481-0

**Published:** 2022-04-07

**Authors:** Yu Hu, Rong Li, Hongyin Chen, Lihao Chen, Xuyue Zhou, Linxi Liu, Mei Ju, Kun Chen, Dan Huang

**Affiliations:** 1 Department of Physiotherapy, Institute of Dermatology, Chinese Academy of Medical Sciences, Peking Union Medical College, 210042 Nanjing, China; 2 Department of Physiotherapy, Jiangsu Key Laboratory of Molecular Biology for Skin Diseases and STIs, Institute of Dermatology, Chinese Academy of Medical Science and Peking Union Medical College, 12 Jiangwangmiao St, 210042 Nanjing, China

**Keywords:** Cutaneous squamous cell carcinoma, Long non-coding RNA, Gene regulatory networks, Microarray analysis, Genome

## Abstract

**Background:**

Cutaneous squamous cell carcinoma (cSCC) is the second most common type of skin cancer, the prognosis for patients with metastatic cSCC remains relatively poor. Thus, there is an urgent need to identify new diagnostic, prognostic, and therapeutic targets and pathways in cSCC.

**Results:**

It detected a total of 37,507 lncRNA probes and 32,825 mRNA probes and found 3593 differentially expressed lncRNAs and 3236 differentially expressed mRNAs. It has been found that mRNAs ACY3, NR1D1, MZB1 has co-expression relationship with six lncRNAs, GXYLT1P3, LINC00348, LOC101928131, A-33-p3340852, A-21-p0003442 and LOC644838.

**Conclusions:**

The aim of this study is to identify cSCC-specific lncRNAs and indicated that six unstudied lncRNAs may serve an important role in endoplasmic reticulum stress apoptosis, autophagy and the progression of cSCC by modulating ACY3, NR1D1 and MZB1.

**Supplementary Information:**

The online version contains supplementary material available at 10.1186/s12864-022-08481-0.

## Introduction

Cutaneous squamous cell carcinoma (cSCC) is the second most common type of skin cancer, accounting for 20% of nonmelanoma skin cancers It is reported that approximately 1.8 million incidences of cSCC in a global context in 2017. The etiology of cSCC is multifactorial, including environmental, immunological, and genetic factors. Of all these risk factors, the most important is cumulative ultraviolet radiation (UVR) exposure [1]. There were still over 2.1% of cSCC patients that developed into lymphatic metastases [2]. Although clinical features and dermoscopic findings could strongly suggest the diagnosis of cSCC, it is still necessary to get a pathological examination to confirm the diagnosis [3]. Moreover, there have no specific biomarkers for the diagnosis of cSCC. The therapeutic options for cSCC includes surgical excision, radiation therapy, chemotherapy and immunotherapy [4]. However, the prognosis for patients with metastasis cSCC remains relatively poor. Thus, there is an urgent need to identify new diagnostic, prognostic, and therapeutic targets and pathways in cSCC.

Long non-coding RNAs (lncRNAs) are a novel class of RNA molecules containing more than 200 nucleotides which have little or no protein-coding capability [5]. LncRNAs are involved in gene transcription and post-transcriptional translation by mediating target gene activation. Previous studies suggest that lncRNAs play critical roles in numerous biological processes, including epigenetic regulation, cell apoptosis cell cycle and cell differentiation regulation [6]. Recently, much attention has focused on the role of lncRNAs in cancer since it could affect various aspects of carcinogenesis, including cancer proliferation, invasion, metastasis and prognosis [2]. A recent review has reported that several dysregulation of lncRNAs play an vital part in cSCC [3]. The expression of *HOTAIR*, *PICSAR*, *LINC00319*, *THOR*, *MALAT1* and *LINC10148* was upregulated in cSCCs and the expression of *GAS5, TINCR* and *LINC00520* was downregulated in cSCCs [7, 8]. However, these certain lncRNAs were mostly identified in in vitro or vivo studies and few lncRNAs studies clarified their roles in cSCC. The role of lncRNAs and their overall contributions to the pathogenesis of cSCC are still unknown. Thus, it is of great importance to explore the potential biological effects of specific lncRNAs on cSCC, which could be used as biomarker for early diagnosis and prognosis prediction.

In order to explore the expression profiles of dysregulated lncRNAs in cSCC and to reveal the potential roles of lncRNAs in the pathogenesis of cSCC, we performed microarray analysis to identify dysregulated lncRNAs and mRNAs. Also, we intended to construct a lncRNA-mRNA correlation network for cSCC, which helps to establish bridges between lncRNAs and mRNAs to reveal the potential functional involvement of lncRNAs in cSCC pathobiology. Furthermore, the expressions of these differentially expressed lncRNAs are verified by quantitative real-time PCR (qRT-PCR). The aim of this study is to identify cSCC-specific lncRNAs and to clarify the molecular mechanism of cSCC, which could provide potential biomarkers and therapeutic targets for cSCC.

## Materials and methods

### Samples

Primary carcinoma tissues and adjacent tissues were obtained from six patients with cutaneous squamous cell carcinoma who underwent surgical treatment at Institute of Dermatology, Chinese Academy of Medical Sciences. All samples from patients were pathologically diagnosed as cSCC and stored in RNA later at -80℃ until RNA extraction. Besides, six normal skin tissues were obtained from six healthy individuals. The study protocol was approved by the ethics committee of the Institute of Dermatology, Chinese Academy of Medical Sciences. All participants had signed written informed consent prior to recruitment.

### RNA extraction and quality control

Total RNA was extracted from the cSCC samples and normal skin tissues using a RNeasy Mini Kit (Qiagen, Hilden, Germany) according to the manufacturer’s protocol. RNA purity and concentration were quantified through Nano Drop ND-1000 (Thermo Fisher Scientific, Waltham, MA) and RNA integrity was assessed by denaturing agarose gel electrophoresis for quality control.

### RNA labeling and hybridization

The lncRNA Human Gene Expression Microarray V4.0 (CapitalBio Corp, Beijing, China) was applied for this detection. The detection starts with the total RNA and performs amplification and fluorescent labeling in vitro. In brief, the First Strand cDNA was synthesized from total RNA and T7 Oligo(dT)Primer (containing a T7 RNA polymerase promoter sequence) and T7-specific primers by using First Strand Enzyme Mix. Then the Second Strand DNA was synthesized by converting the RNA strand in the DNA-RNA hybrid into Second Strand cDNA using Second Strand Enzyme Mix. cRNA was synthesized from Second Strand cDNA by using T7 Enzyme Mix. High yields of Cy3- and Cy5-labeled cDNAs were produced using the CapitalBio cRNA Amplification and Labeling Kit (CapitalBio, Beijing, China). The labeled products were used for microarray hybridization.

### Microarray assay

The global profiling of human lncRNA and mRNA expression were performed with the CapitalBio Technology Human lncRNA Array V4 (CapitalBio, Beijing, China). The acquired tiff format images were obtained using Agilent Feature Extraction (V10.7). Quantile normalization, quality control and subsequent data processing were performed using the Agilent GeneSpring software (V13.0). The differentially expressed lncRNAs and mRNAs were identified via Volcano plot filtering with the threshold set of fold change ≥2.0 and *P*-value < 0.05. Further hierarchical clustering analysis was performed to present lncRNA and mRNA expression patterns.

### Gene ontology and pathway enrichment analysis

Gene ontology (GO) analysis (http://www.geneontology.org) is frequently used in functional enrichment studies of large-scale genes and categorizes the roles of mRNAs into three domains, including biological process, cellular component and molecular function. GO analysis was performed to identify the potential functions of differentially expressed genes. Pathway analysis was applied to reveal potential biological pathways associated with differentially expressed genes according to the KEGG (Kyoto Encyclopedia of Genes and Genomes) database (http://www.genome.ad.jp/kegg/).

### LncRNA-mRNA correlation analysis

To reveal the association between the lncRNAs with direct regulated expression of target mRNAs, we conducted the co-expression analysis. The co-expressed lncRNAs and mRNAs were selected with the standard of Pearson correlation > 0.99 or < −0.99 and *P* value < 0.05. The top 1000 gene pairs were used to construct the coding-non-coding gene co-expression network using bioinformatics Cytoscape software.

### Target prediction

Target prediction can be divided into cis-prediction and trans-prediction. Cis-prediction could predict the co-expressed lncRNAs and mRNAs through the positional comparison of lncRNAs and mRNAs. Trans-prediction could predict the possible relationships through sequence alignment. Based on the results of the correlation analysis of lncRNA and mRNA (Correlation>0.99 or Correlation<-0.99, and *P* value<0.05), cis-prediction looks for lncRNA-mRNA pairs whose genome position is within 10 kb. Trans-prediction was conducted using blat tool to select lncRNA-mRNA pairs with similar sequences by comparing lncRNA and mRNA (3’UTR) sequences.

### Quantitative RT-PCR

Complementary DNA was synthesized using the Reverse Transcription Kit (Takara, China). qRT-PCR was performed using the SYBR Green One Step qPCR Kit (Biotool) on the ABI 7500 Real-Time PCR machine (Applied Biosystems). Primers used for qPT-PCR are listed in Table 1. For quantitative results, 2 − ΔΔCt method was used to calculate the relative expression level of each lncRNA.

**Table 1:** Primers used for reverse transcription-quantitative PCR

lncRNA	Forward primer (5′→3′)	Reverse primer (5′→3′)
PVT1	TGAGAACTGTCCTTACGTGACC	AGAGCACCAAGACTGGCTCT
CTD-2521M24.9	CTCGTTCTTAGGCAGCATCTGTGTC	TGGAGACTGAAAGGTGGTGTTGATTG
AL353997.3	GTGAAACCTCAGATGCCCATTTGTAAC	CCTCTTTGGTGCCTGGTGCTATG
MIR4720	GGAACCTGGCACCACACTAAACC	GGGAATGAACACGAATACGGAATAAGC
BX004987.5	CATCCTGGCTAACTCAGTGAAACCC	CACACCATTATCCTGCCTCAGTCTC
CTD-2619J13.13	CAATGTCTGCCTGCCTATCCACTG	GAAGAGAAAGAGGAGATTGGCTGAGG
LINC00478	TGCCTTAACTGATGACATTCCACCAC	AGCCAGGATGAGCCAGGAGAAG
GAPDH	TGCCTTAACTGATGACATTCCACCAC	AGCCAGGATGAGCCAGGAGAAG

*lncRNA* long non-coding RNA

### Statistical analysis

All statistical analyses were performed using the SPSS version 17.0 software (SPSS, Inc., Chicago, USA). Data were analyzed by two-tailed Student’s t test. *P*<0.05 was considered as a statistically significant difference.

## Results

### Identification of differentially expressed lncRNAs and mRNAs in cSCC

**Fig. 1:**
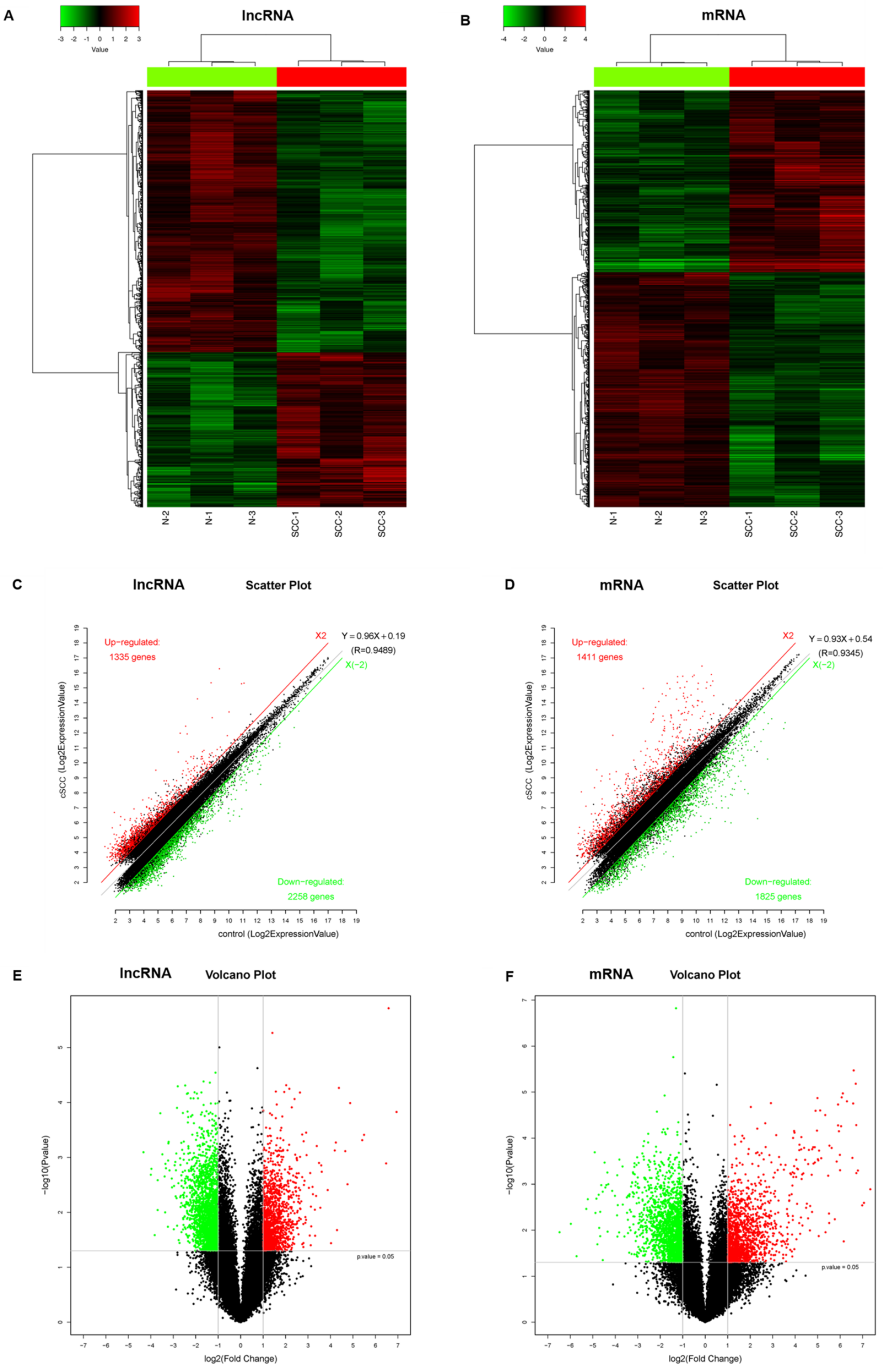
Differentially expressed lncRNAs and mRNAs in CSCC. The expression of lncRNAs and mRNAs were compared between CSCC and normal cutaneous tissue specimens. Hierarchical Clustering was applied to group lncRNAs and mRNAs based on their expression levels (**A** and **B**). The horizontal axis represents fold of change in expression (on a log2 scale) and the vertical axis represents *P*-value (on a negative log10 scale). The scatter plot and volcano plot of lncRNAs (**C** and **E**) and mRNAs (**D**, **F**) illustrate the difference in the expression levels of each transcript. The red and green dots represent transcripts significantly upregulated and downregulated in cSCC, respectively. lncRNA, long non-coding RNA; cSCC, cutaneous squamous cell carcinoma

The microarray analysis revealed that the global profiling of differentially expressed lncRNAs and mRNAs between cSCC and healthy control. From the lncRNA expression profiles, 3593 differentially expressed lncRNAs and 3236 differentially expressed mRNAs were performed with the threshold of fold-change ≥2.0 and *P*≤ 0.05, with the results are shown in Table S 1. Hierarchical Clustering was applied to group lncRNAs and mRNAs based on their expression levels (Fig. 1A and B). Among all the differentially expressed LncRNAs and mRNAs, 1335 lncRNAs and 1411 mRNAs were upregulated and 2258 lncRNAs and 1825 mRNAs were downregulated in cSCC cancer tissues compared to normal skin tissues. The scatter plot and volcano plot of these probe-matched gene expressions of LncRNA and mRNA shows the up-regulated and down-regulated lncRNAs (Fig. 1C and E) and mRNAs (Fig. 1D, F) profiling across groups. The red dots and green dots stand for up-regulated and down-regulated expressions, respectively.

### Gene ontology and pathway analyses

**Fig. 2:**
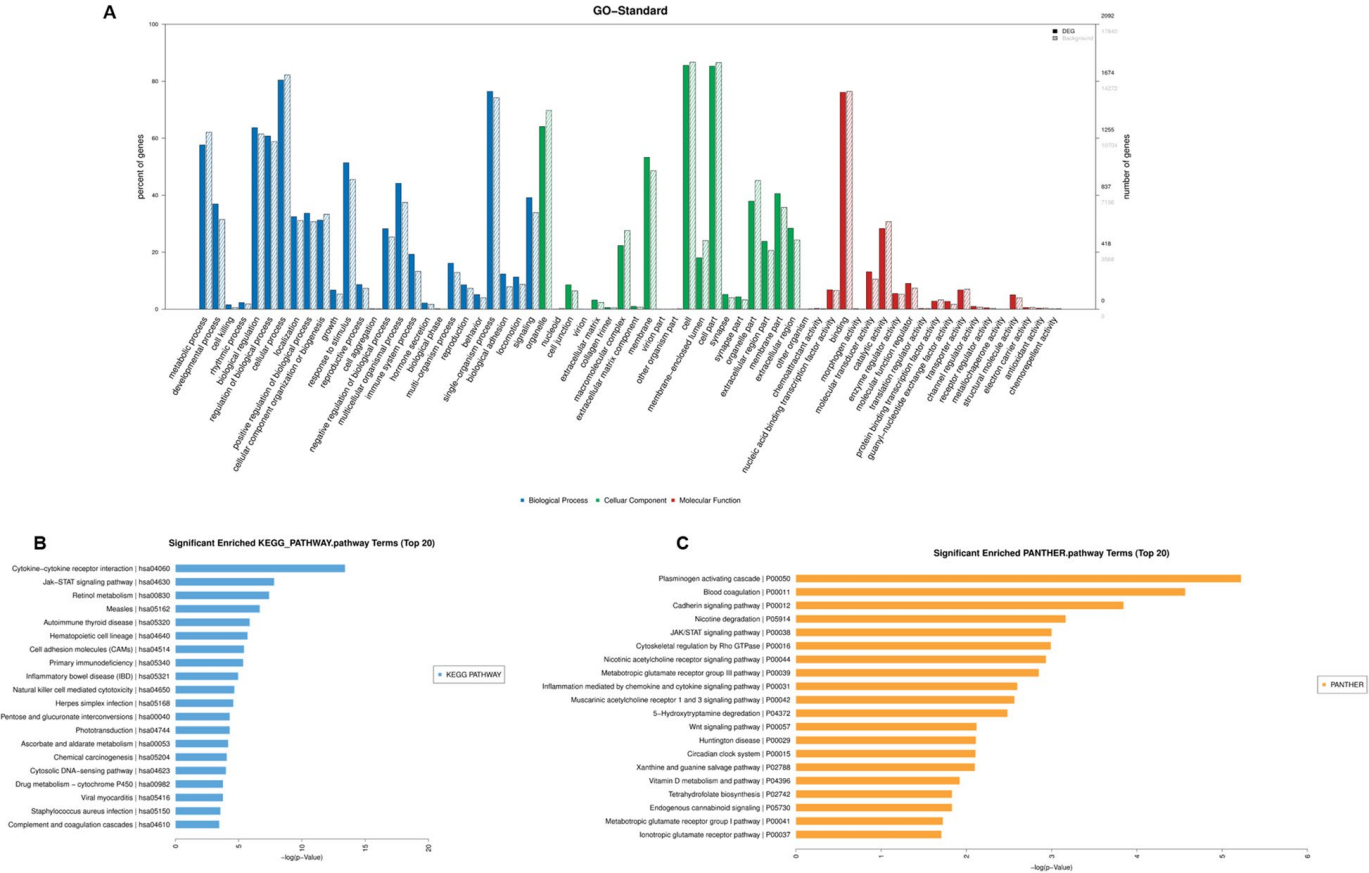
Gene ontology and KEGG pathway analyses. **A** Top 20 GO terms with significantly differential expression from GO analysis are categorized into biological process (blue), cellular component (green) and molecular function (red). **B** Top20 KEGG pathways with significantly differential expression are shown. **C** Top20 PANTHER pathways with significantly differential expression are shown. Vertical axis represents LgP values indicate significance of the enrichment. GO, Gene Ontology; KEGG, Kyoto Encyclopedia of Genes and Genomes; lncRNA, long non-coding RNA; cSCC, cutaneous squamous cell carcinoma

GO analysis was conducted to classify differentially expressed mRNA into three categories, including biological processes, cellular components and molecular function. Among the involved biological processes, cellular process, single-organism process, biological regulation, regulation of biological processes and metabolic processes, were the five most significant processes associated with the dysregulated mRNAs. Among the cellular components, cell, cell parts and organelle were the three most significant component processes associated with the dysregulated mRNAs. Among molecular functions, binding, catalytic activity and molecular function regulator were the three most significant molecular functions of the dysregulated mRNAs (Fig. 2A).

Pathway analysis was carried out based on the KEGG, BioCyc and Panther database. The top 20 enrichment score values of the enriched pathways by KEGG pathway (Fig. 2B) analysis and Panther pathway analysis (Fig. 2 C). The dysregulated mRNAs were most associated with “Cytokine-cytokine receptor interaction”, “Jak-STAT signaling pathway” and “Retinol metabolism” in KEGG pathway. The dysregulated mRNAs were most associated with “Plasminogen activating cascade”, “Blood coagulation”, “Cadherin signaling pathway”, “Nicotine degradation” and “Jak-STAT signaling pathway” in Panther pathway.

### LncRNA-mRNA co-expression network

**Fig. 3:**
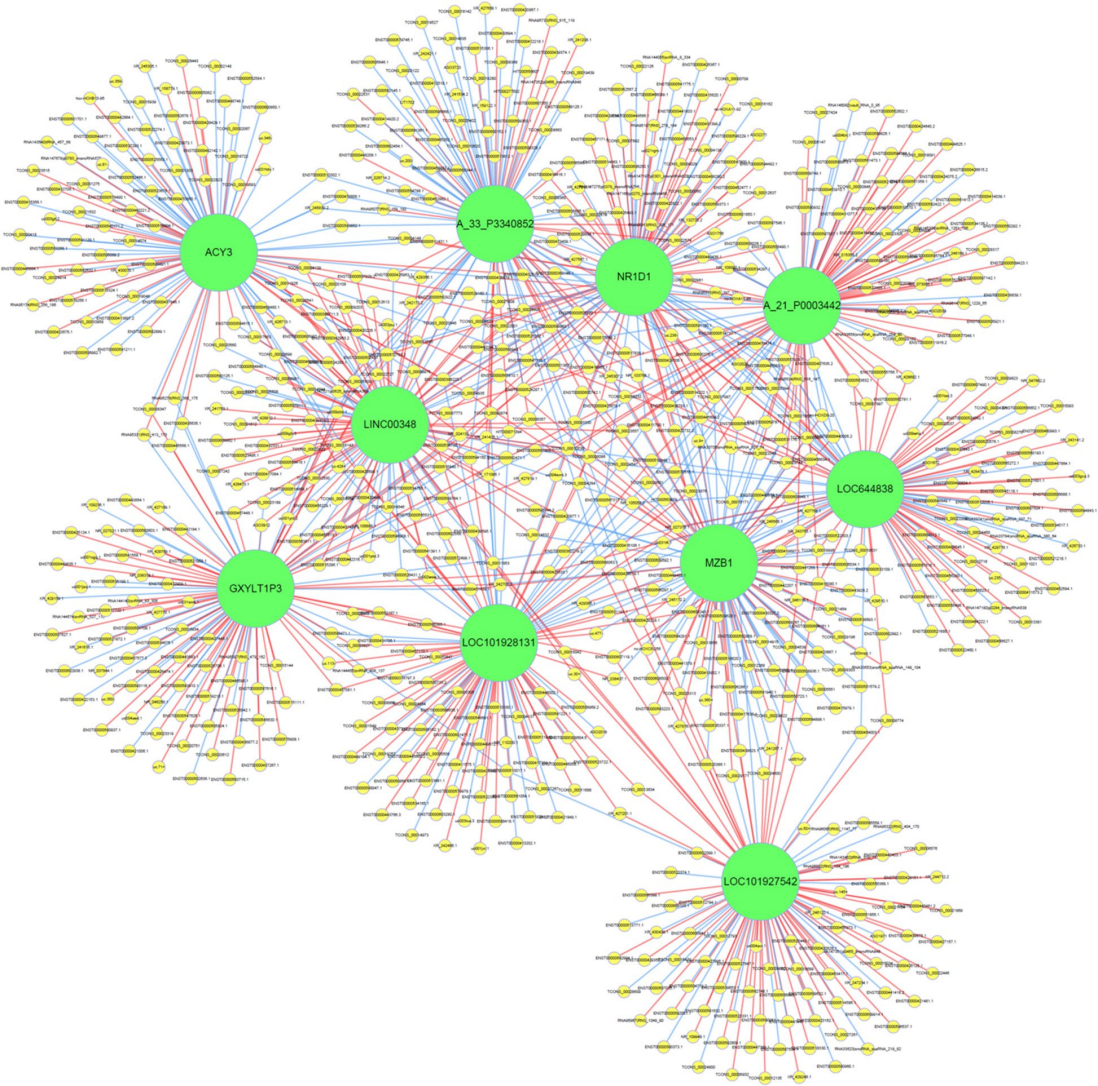
Construction of the lncRNA-mRNA co-expression network. Green nodes represented for LncRNA, yellow rodes represent for the correlated mRNA, each circle’s size indicates relative number of related genes. The blue lines indicate negative correlations and the red lines are positive correlations

We first constructed genes co-expression networks between the cSCC group and normal skin group with the all differential lncRNAs and mRNAs in this study. Among these, the lncRNAs and mRNAs were selected with the threshold set of Pearson’s correlation coefficients >0.99 or <-0.99 and *P*-value <0.05. Subsequently, the top 1000 enriched gene pairs of lncRNAs and mRNAs, correlation coefficient was constructed to build the co-expression network by using cytoscape program, as shown in Fig. 3. In the co-expression network, A_33_P3340852 was obtained the highest number of interactions.

### The target mRNA of LncRNA prediction and functional analysis

**Fig. 4:**
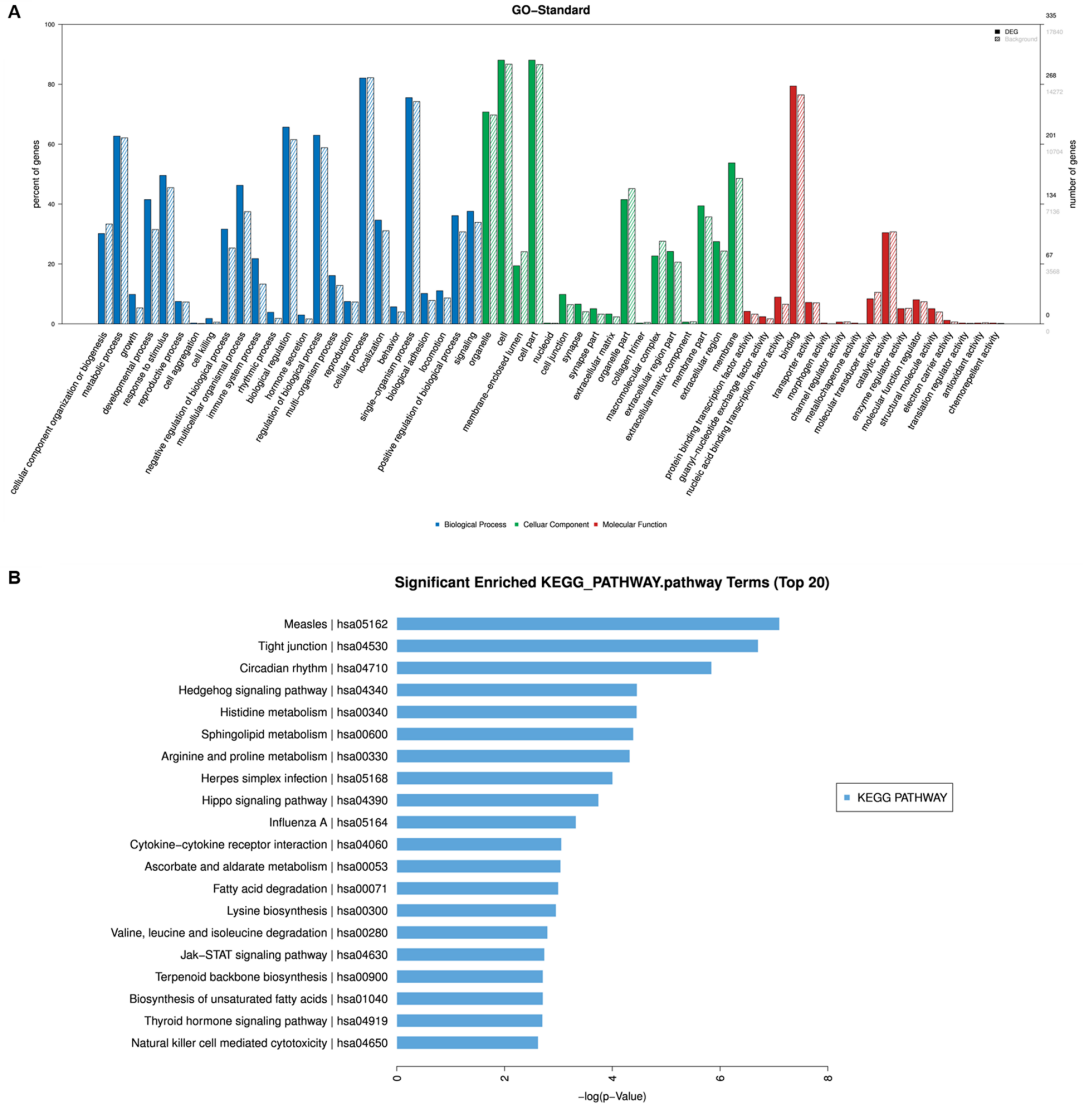
The target mRNA of LncRNA prediction and functional analysis. **A** Top 20 GO terms of the targeted mRNA with significantly differential expression from GO analysis are categorized into biological process (blue), cellular component (green) and molecular function (red). **B** Top20 KEGG pathways of the targeted mRNA with significantly differential expression are shown. Vertical axis represents LgP values indicate significance of the enrichment.GO, Gene Ontology; KEGG, Kyoto Encyclopedia of Genes and Genomes; lncRNA, long non-coding RNA; CSCC, cutaneous squamous cell carcinoma

To investigate whether the differentially expressed lncRNAs regulate genes and determine the signaling pathways associated with cSCC, target prediction was performed to predict the possible targets of the dysregulated lncRNAs. Based on the results of the co-expression network of lncRNAs and mRNAs (Correlation>0.99 or Correlation<-0.99, and P < 0.05), cis-prediction looks for lncRNA-mRNA pairs whose genome position is within 10 kb, and trans-prediction compares lncRNA and mRNA (3’UTR) sequences, and selects lncRNA-mRNA pairs with similar sequences. The target genes of lncRNAs with top 1000 correlation coefficient were shown in Fig. 4.

Then we performed GO enrichment and KEGG pathway analyses on the target mRNAs (Fig. 4). Through GO analysis, we found that the target expressed mRNAs were enriched for cellular process, single-organism process, biological regulation linked with biological processes, and cell, cell part, organelle involved in cellular components, as well as binding, catalytic activity, nucleic acid binding transcription factor activity in molecular functions (Fig. 4A). For the KEGG pathway analysis, the top 20 enrichment score values of the enriched pathways included measles, tight junction, circadian rhythm, hedgehog signaling pathway and histidine metabolism (Fig. 4B).

### RT-qPCR validation of differentially expressed lncRNAs

**Fig. 5:**
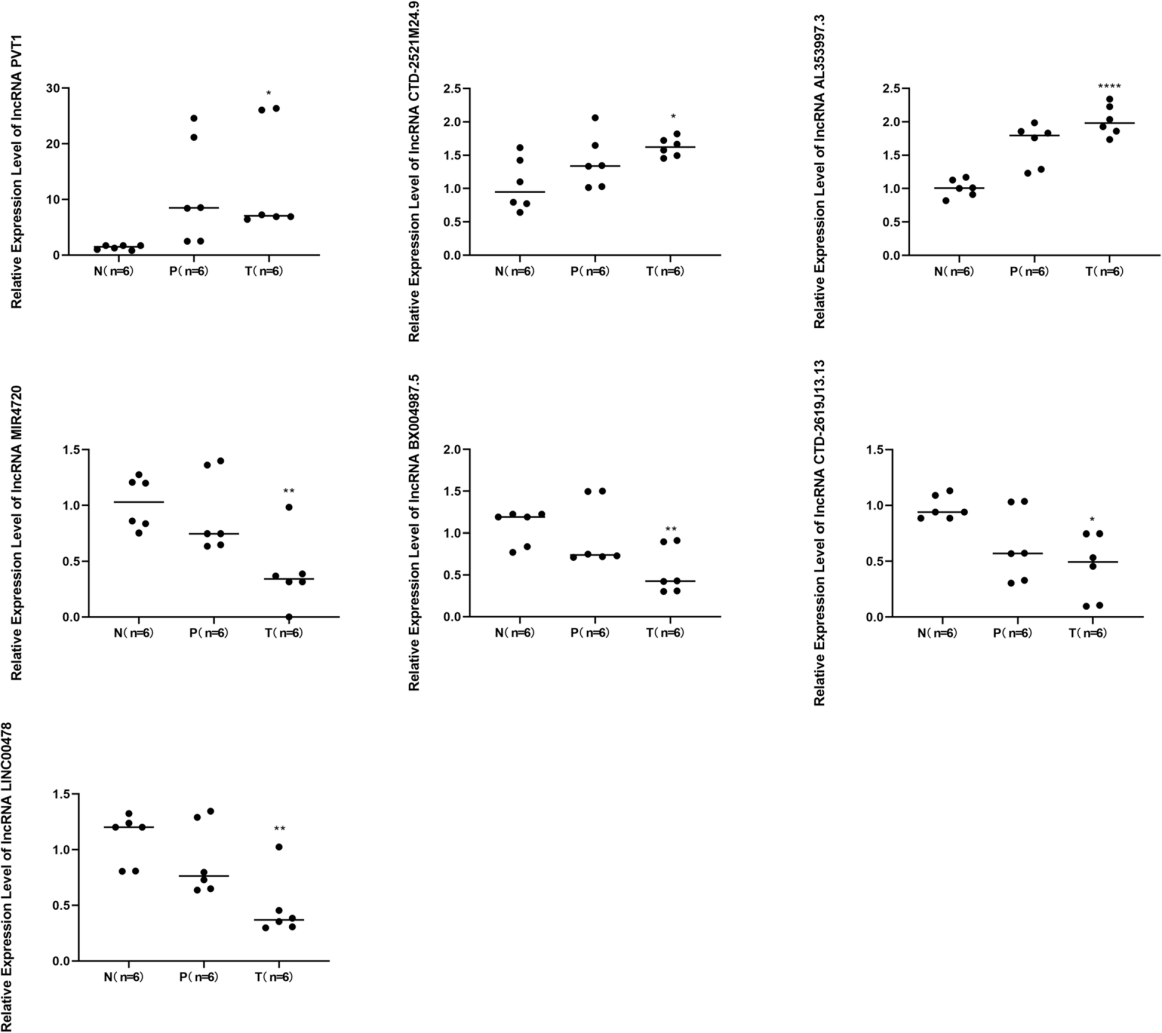
Validation of the expression lncRNAs by qRT-PCR. The expression of top 7 differentially lncRNAs are compared between six paired cSCC tissues ,matched adjacent and normal tissue samples. There are significant difference between cSCC tissues and normol tissue. *P < 0.05 ,**P < 0.01 and ****P < 0.0001. RT-qPCR, reverse transcription-quantitative PCR; N, distant normal cutaneous tissue ; P, para-tumor tissue; T, tumor tissue

The top 7 DE lncRNAs (Table 1) were selected for RT-qPCR analysis to verify the microarray results in six paired cSCC tissues, matched adjacent and normal tissue samples. The RT-qPCR results demonstrated that three lncRNAs (PVT1,CTD-2521M24.9 and AL353997.3) were upregulated in cSCC and four lncRNAs (MIR4720,BX004987.5,CTD-2619J13.13andLINC00478) were downregulated in cSCC. As shown in Fig. 5, the relative values of the expression levels detected by RT-qPCR were found to be consistent with the microarray data. This result suggests that the transcript identification and abundance estimates were highly reliable.

## Discussion

Recent studies have revealed the significant role of lncRNA in various types of cancer for tumorigenesis and development, such as non-small-cell lung cancer, liver cancer, bladder cancer and breast cancer [9–11].Moreover, numerous lncRNAs play an important role in the onset and progression of diseases by affecting cell proliferation ,invasion and other functions [12]. As the second most common cancer worldwide ,cSCC has been reported with an annual accidence with over one million individuals [13–15]. UV irradiation, particularly UVB, is one of the classical cellular stressors causing DNA damage in skins, which receives chronic sun exposure, and ultimately leading to skin development of cSCC [16]. Therefore, many present studies investigated the potential oncogenic role of molecules and pathways in cSCC and tried to evaluate the promising diagnostic biomarkers value for cSCC [17–20]. It is currently aimed to elucidate DNA binding/differentiation 4 (ID4) function in cSCC development [21]. The results indicate that UVB irradiation leads to abnormal downregulated ID4 via DNA methylation and ID4 acts as a tumor suppressor gene in tumorigenesis. In addition, reports indicates that the phosphoinositide3-kinases (PI3Ks)/Akt pathway plays an essential role in advanced cSCC, which inhibit viability and growth of cSCC and can also prove to be valid target in cSCC [22]. Some dysregulated transcription factors (MYC, RELA, ETS1, SP1, TP63, TP53, AP1, TCF3, SOX2, OCT-3/4) and downregulated epidermal differentiation genes (LCE1D, FLG, KRT77, KRT10, ALOEX3 etc.) are all altered at the transcript level in cSCC [23–26]. However, the underlying molecular mechanism of LncRNAs in cSCC are rarely mentioned and still remains unclear.

To investigate whether lncRNA is involved in the carcinogenic process of the development of cSCC, we performed lncRNA microarray to compare the comprehensive lncRNA expression profiles in three paired cSCC tissues and normal skin samples. In this study, through Hierarchical Clustering, we identified differential expression of lncRNAs and mRNAs in cSCC tissues and predicted the potential biological function, cellular progress and enriched pathways by GO, KEGG and Path-net analysis. It detected a total of 37,507 lncRNA probes and 32,825 mRNA probes and found 3593 differentially expressed lncRNAs and 3236 differentially expressed mRNAs. The selection criteria, fold change >2 and *P* < 0.05, ensured the significance of the differential expression data. Among these differentially expression lncRNAs, the top 7 lncRNAs, including four downregulated lncRNAs and three upregulated lncRNAs, were confirmed expressions by real-time PCR. Results of the qRT-PCR were consistent with those trends of high-throughput sequencing, proving its reliability. Of these verified lncRNAs in the present report, some have been identified as oncogenic lncRNA in carcinogenesis. For instance, the p29508 probe detected lncRNA PVT1, a top overexpressed lncRNA in microarray analysis [27]. Accumulating evidence suggests that the *PVT1* locus as an epigenetic enhancer in colorectal cancer (CRC) and it has a regulatory effect on regulating the expression of MYC. Furthermore, PVT1 lncRNA expression mediated through aberrant methylation may also impact TGFβ/SMAD and Wnt/β-Catenin pathways by the key CRC gene. Thus, high expression of the PVT1 lncRNA may serve a pivotal role in the pathogenesis of CRC and has demonstrated roles in several aspects, as a prognostic biomarker and a potential therapeutic target. Moreover, upregulated PVT1 can activate Wnt/β-catenin signaling pathway by regulating expression of both Pygo2 and ATG14 and thus promote autophagy related complex in pancreatic cancer [28]. Similarly,it has already been reported in anothor study that lncRNA PVT1 inhibite the proliferation of gastric cancer by combining with enhancer of zeste homolog 2 (EZH2) to repress p15 and p16 [29]. Thus, we hypothesize that lncRNA PVT1 upregulation may serve as an oncogenic gene in cSCC by combing EZH2 to activate Wnt/β-catenin pathways and promote tumorigenic process of proliferation, autophagy and apoptosis aspects. Besides, LINC00478 has been identified to be highly link to accurate classification of subtypes of breast cancer by facilitating expression of relevant miRNA profiles [30].

LncRNA is a class of nucleotides that transcription length are more than 200 nt and which have little or no protein-coding capability [5, 31, 32]. These molecules play crucial roles in several aspects of biological functions as chromatin remodeling, gene transcription level regulation and protein modification during the occurrence and development of many diseases [33, 34]. An increasing number of studies described that dysregulated lncRNA expression is associated with various cancers and may contribute to tumorigenesis, metastasis and prognosis of many tumors [35]. Recent studies demonstrated that lncRNAs can be utilized as a disease biomarkers and biomarkers for survival prediction in cervical cancer, colorectal cancer and skin cancers [36–38].Available evidence has reported that comprehensive analysis of interaction between lncRNAs and mRNAs may provide a reference for further explore its biological functions and the potential underlying mechanisms in cancers [39]. In order to better study whether lncRNA is involved in cSCC tumorigenesis, we constructed co-expression network of top 1000 pairs of lncRNAs and mRNAs to annotate clear biological functions and regulatory mechanism of lncRNAs [40]. After construction of the co-expression network, it has been found that mRNAs ACY3, NR1D1, MZB1 has co-expression relationship with six lncRNAs, GXYLT1P3, LINC00348, LOC101928131, A-33-p3340852, A-21-p0003442 and LOC644838 which largely have not been studied before. Otherwise, NR1D1is reported to showed significant correlation with regulation of autophagy activities by microphages, metabolic process of stress response, signal transduction and inflammatory pathways [41, 42]. The results of study suggested that MZB1 expression plays an critical role in advanced stage of breast cancer and may be a poor prognostic marker,which interact with endoplasmic reticulum stress related pathways [43]. ACY3 has rarely been reported but also may contribute to pathogenesis of Huntington disease (HD) by altering binding of transcriptional factors [44]. Within the co-expression network, that NR1D1 was noted that are connected with the maximum number (six) of lncRNAs, indicating that it may play a significant role in the development and progression of cSCC. The dysregulation of these three transcripts mainly contribute to modulating apoptosis induced by endoplasmic reticulum stress, physiological processes of cellular signal transduction and metabolic regulation, inflammatory signaling pathways and autophagy.

In the present study ,the enrichment analysis of GO,KEGG and Panther pathway identified that“Cytokine-cytokine receptor interaction”, “Jak-STAT signaling pathway”,“Plasminogen activating cascade ”and “Cadherin signaling pathway” were the top four significantly changed pathways. It has been reported that Jak-STAT signaling pathway can contribute to resistance of cervical squamous cell carcinoma by mediating key activator protein as STAT1/STAT2 [19]. Jak-STAT signaling pathway inhibition is involved in anti-tumor activity in several pre-clinical studies and continued activation of STAT1 and STAT2,which are essential components of JAK/STAT pathway, are associated with suppressing tumor apoptosis [45, 46]. Meanwhile, the present study found that exogenous IL-6 can induce activation of JAK/STAT signaling and to increase autophagy [47]. Furthermore, Cadherin signaling pathway can act as an intracellular signal transducer and was involved in invasion and metastasis by Wnt/β-catenin pathways inducing EMT expression profiles in tumor progression [48].

In fact, four of significant enriched pathways, including the“Cytokine-cytokine receptor interaction”, “Jak-STAT signaling pathway”,“Plasminogen activating cascade” and “Cadherin signaling pathway”, were coincided with the pathogenesis mechanism of co-expression network ,which mainly were focused on endoplasmic reticulum stress of apoptosis ,cellular signal transduction and autophagy in tumorgenesis, as aforementioned. Since each results were individually analyzed and relationships of co-expressed lncRNA-mRNA and pathway analysis are independent, this outcome greatly supports the reliability of the current study. Therefore, it was hypothesized that six lncRNAs, GXYLT1P3, LINC00348, LOC101928131, A-33-p3340852, A-21-p0003442 and LOC644838, in the co-expression network may serve a considerable role in the progression of cSCC by regulating apoptosis induced by endoplasmic reticulum stress, cellular signal transduction and autophagy.

In conclusion, the present study revealed a comprehensive analysis of lncRNA-mRNA co-expression profiles of patients with cutaneous squamous cell carcinoma. GO, KEGG and PANTHER analyses provided the function of mRNAs and suggested the possible biological effects of lncRNAs. The results indicated that six lncRNAs, which have not been studied in cSCC,may serve an important role in endoplasmic reticulum stress apoptosis, autophagy and the progression of cSCC by modulating ACY3, NR1D1 and MZB1. However, there are still some defects which need to be improved to carry out more comprehensive research, including small study size containing only six tissues and the lack of experimental verification in liquid biopsies (plasma/serum)of cSCC.These data argue for further intensive research to provide a more comprehensive understanding of the molecular mechanisms underlying the metabolism of cSCC.

## Supplementary Information

**Additional file 1.**

**Additional file 2.**

## Data Availability

All data generated or analyzed during this study are included in this published article.
